# Supplementary material for: Loss of foxo rescues stem cell aging in Drosophila germ line
Source: eLife. 2017 Sep 19;6:e27842. doi: 10.7554/eLife.27842 (PMC5644957; doi:10.7554/eLife.27842)
Supplement: Supplementary file 1. — (A) Table showing the number of GSCs with elongated spectrosomes expressed as mean percent values (±SE). (B) Table showing the number of germaria with branched fusomes expressed as mean percent values (±SE). [file elife-27842-supp1.pdf]

A % GSCs w/ Elongated Spectrosomes

	Unirr.	1 day post-IR	2 days post-IR
**UAS-Dcr-2; nos-Gal4***	**26 (± 2.56)%**	**3 (± 0.5)%**	**14 (± 2.27)%**
nos-Gal4>Foxo RNAi 1	16 (± 1.2)%	10 (± 0.18)%	12 (± 1.17)%
nos-Gal4>Foxo RNAi 2	15%	12%	---
Foxo RNAi 1**	24 (± 1.26)%	3 (± 0.7)%	15 (± 0.73)%
nos-Gal4>Tor RNAi	16 (± 3.12)%	1 (± 0.85)%	6 (± 1.72)%
nos-Gal4>Thor RNAi	17%	4%	14%
nos-Gal4>Loki RNAi	12%	26%	---
nos-Gal4>BL 43962***	17%	---	11%

***Uas-Dcr-2; nos-Gal4 = control**
**Foxo RNAi 1 = Foxo RNAi construct only, no Gal4
***nos-Gal4>BL 43962 = additional RNAi control line

B % Germaria w/ > 4 progeny or ≥ 1 branched fusome

	Unirr.	1 day post-IR	2 days post-IR
**UAS-Dcr-2; nos-Gal4***	**99 (± 0.71)%**	**17 (± 6.17)%**	**5 (± 2.95)%**
nos-Gal4>Foxo RNAi 1	99 (± 5.88)%	85 (± 6.16)%	22 (± 6.16)%
nos-Gal4>Foxo RNAi 2	100%	68%	---
nos-Gal4>Tor RNAi	100 (± 0)%	9 (± 4.17)%	2 (± 2.14)%
nos-Gal4>Loki RNAi	95%	77%	---

***Uas-Dcr-2; nos-Gal4 = control**
**Foxo RNAi 1 = Foxo RNAi construct only, no Gal4
***nos-Gal4>BL 43962 = additional RNAi control line
